# Laparoscopic light source skin burn

**DOI:** 10.1093/jscr/rjae116

**Published:** 2024-03-07

**Authors:** Jamal T Hamdi

**Affiliations:** Surgical Department, Medical College, Umm Al-Qura University, Makkah, Saudi Arabia

**Keywords:** laparoscopic surgery, light source, skin burn, endoscopy light, theater hazard

## Abstract

Skin burn injury from light cables is a rare complication of laparoscopic surgery, but it can be severe and distressing for both the patient and the surgeon. A case report of skin burns due to laparoscopic light source is presented in this article, followed by an experimental trial to confirm the findings, and review of literature. The light source is usually connected to the camera to give adequate light inside the abdominal cavity for visualization of the internal organs, and hence, safe surgery. The light source should deliver cool light to prevent any burn from heat to skin or internal organs, but in fact, it is not usually cool and can reach high temperatures. Precautions and recommendations to avoid skin burns due to the light source are included. Surgeons should be aware of burns from light sources in laparoscopic surgery and take precautions to prevent them.

## Introduction

Light cable skin burn is a rare complication of laparoscopic surgery, and it is usually silent, but it can be severe and distressing for the patient and the surgeon. Recent systematic literature review identified only seven articles related to microsurgery-related cutaneous burns [1]. Cutaneous burns during knee arthroscopy are also rare, as reported in previous case report in this journal [2], and the national review from UK identified only seven cases over 15 years [3]. An experimental study with rat skin exposed to operating microscope lights showed that, by using direct exposure of the microscope light to a thermometer probe, the temperature reached in excess of 80°C when 100% xenon light was applied from a distance of 200 mm [4]. Another study showed that burying the end of the cable within the paper surgical drape produced a hole within 15 s, and in the cotton towel, it produced a yellow discoloration after 2 min [5]. Various microscope-dependent factors such as focal length, illumination strength, and exposure time may have an effect on iatrogenic thermal burns [6].

Laparoscopic instruments usually undergo vigorous checks to ensure their safety for patients and staff. The light source is usually connected to the camera, and it has many heat filters to reduce heat, and it gives adequate light inside the abdominal cavity for visualization of the internal organs, and hence, safe surgery. The light source should deliver cool light to prevent any burn from heat to skin or internal organs. It is important to be aware that a so-called “cold” light source may produce radiant thermal injury when the fiberoptic cable is applied directly to the skin [7]. A case report of a patient suffered from skin burns due to laparoscopic light source is presented, followed by experimental trial, to confirm the findings, and review of literature.

## Case report

**Figure 1:**
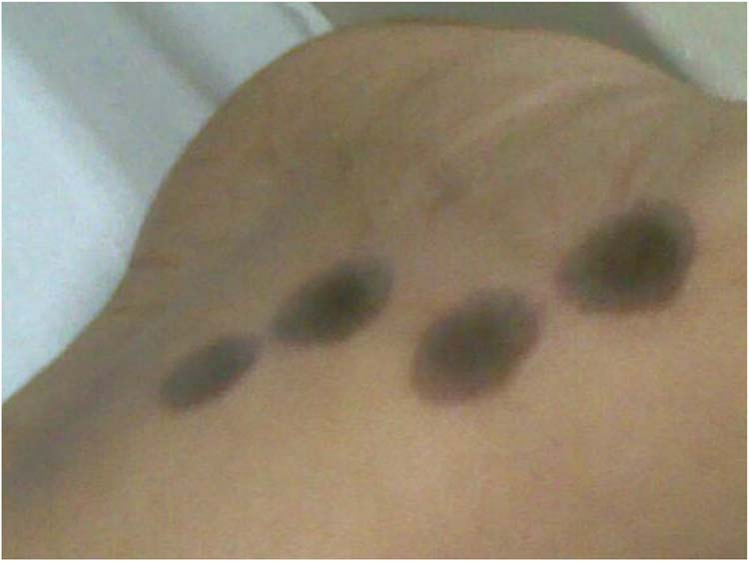
Image showing skin burns.

A 45-year-old female patient who had long-standing chronic cholecystitis had an uneventful laparoscopic cholecystectomy. During recovery from general anesthesia, the patient complained of pain in the right foot. Four rounded skin burns were discovered on the inner side of right foot and it cannot be explained (Fig. 1).

**Figure 2:**
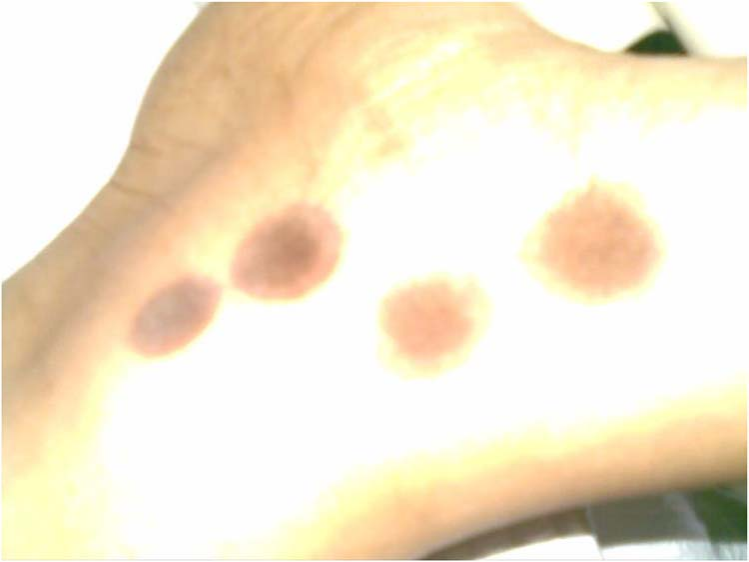
Image showing healing of burn.

The skin burns were treated locally by Flamazine, and 3 days later, improvement was noted (Fig. 2), and the patient was discharged home.

**Figure 3:**
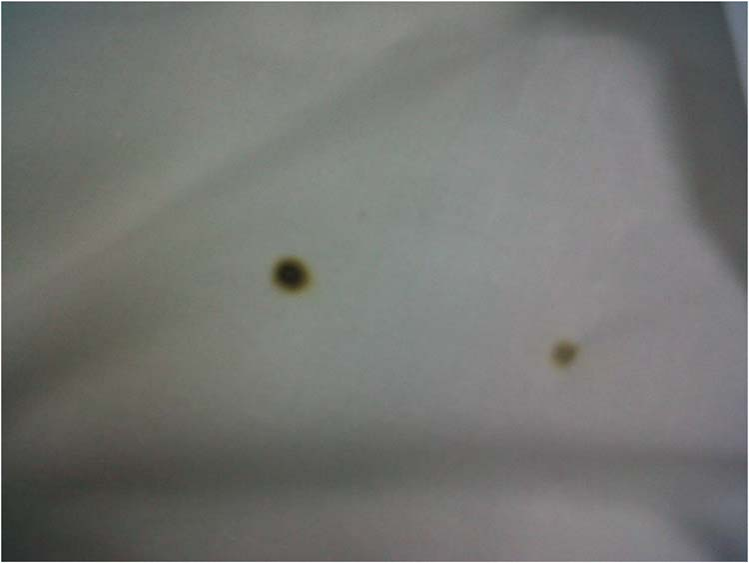
Burn on outside drape.

**Figure 4:**
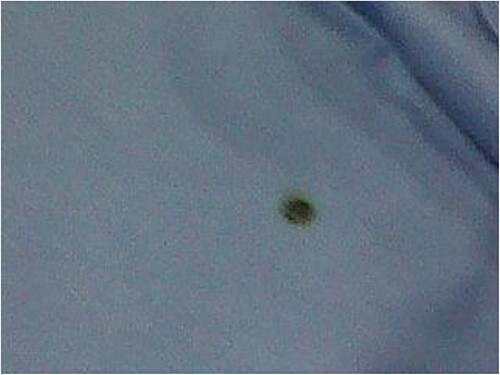
Burn on inside drape.

The burn was suspected to be from the laparoscopic light source. To confirm the suspicion, the light source was tested on two surgical drapes used to cover the patient during surgery. The results are shown on the outside drapes (Fig. 3) and inside drapes (Fig. 4).

These burns were produced quickly from the direct application of light source to drapes. Longer duration of application of light source produced more penetration of the burn, with the risk of fire at any time.

## Discussion

Thermal injury from electrocautery systems has long been shown to be a complication of minimally invasive surgery, and the earliest reports were in the settings of gynecologic laparoscopy and gastrointestinal endoscopy [8], and this should not be mixed with skin burn from light cables. Light sources used in laparoscopy proved capable of burning a hole in a standard paper surgical drape in <5 s [9–11]. Laparoscopic light cables are available as fiberoptic or are gel-filled. The fiberoptic cables transmit the light through internal reflections in tiny fibers. The gel-filled cables consist of a clear liquid optical gel that is capable of transmitting 30% more light than optic fibers, and they can be more hazardous than the fiberoptics since they transmit more heat [12]. Although cloth drapes did take slightly longer time to burn, the short time difference would not matter much in practice. The light source should be switched on only after the light cable has been connected to the scope and must never be left resting on the drapes once the light source has been switched on because thermal burns will occur within seconds. The surgeon should always make sure that the fiberoptic cable tip is not left lying on the patient when it is not in use. To improve patient safety, the light source should be turned off immediately after surgery and the tip should be kept in a safe place. Some surgeons changed the light source to LED and found the heat generated at the tip of the laparoscope reduced, with less risk of burn [13]. Recommendation on the steps to be taken to prevent light source burn have been issued recently by the Quality and Patient Safety Commission [14] and by Insurance Agency to prevent litigation [15] .

## Conclusion

Surgeons who perform laparoscopic or endoscopic procedures should be aware of the potential for patient burns from the light sources associated with these scopes. These burns can be silent and go unnoticed by the surgical team because they typically do not produce smoke or charring.

## Data Availability

Data are available on reasonable request.
